# A note of thanks to referees in 2014

**Published:** 2014

**Dementia & Neuropsychologia** finishes the eighth year of publication and we would like to thank our referees for sharing their expertise with us. The high quality of constructive criticisms and dedication of all our reviewers are highly appreciated by both the editors and the authors.

**Table:** 

Adriana Bastos Conforto (SÃO PAULO, SP) Alessandro Ferrari Jacinto (BOTUCATU, SP) Alexandre Leopold Busse (SÃO PAULO, SP) Alfredo Ardilla (MIAMI, USA) Ana Cristina de Albuquerque Montenegro (RECIFE, PE) Ana Luisa Gomes Pinto Navas (SÃO PAULO, SP) Analuiza Camozzato de Padua (PORTO ALEGRE, RS) Angelina Maria Martins Lino (SÃO PAULO, SP) Antonio Lucio Teixeira Junior (BELO HORIZONTE, MG) Benito Damasceno (CAMPINAS, SP) Brasilia Maria Chiari (SÃO PAULO, SP) Carla Adda (SÃO PAULO, SP) Carla Monteiro Girodo (BELO HORIZONTE, MG) Carlos Ketzoian (MONTEVIDEO, URUGUAY) Carlos Roberto de Mello Rieder (PORTO ALEGRE, RS) Carmem Lisa Jorge (SÃO PAULO, SP) Carol Dillon (BUENOS AIRES, ARGENTINA) Cassio Machado de Campos Bottino (SÃO PAULO, SP) Celia Maria Giachetti (MARÍLIA, SP) Ceres Eloah de Lucena Ferretti (SÃO PAULO, SP) Charles André (RIO DE JANEIRO, RJ) Clara Regina Brandão de Avila (SÃO PAULO, SP) Claudia Maia Memória (SÃO PAULO, SP) Claudia Sellito Porto (SÃO PAULO, SP) Dalva Lucia Rollemberg Poyares (SÃO PAULO, SP) Karin Zazo Ortiz (SÃO PAULO, SP) Karina Pagliarin (PORTO ALEGRE, RS) Karolina Gouvea Cesar (SÃO PAULO, SP) Knut Engedal (OSLO, NORWAY) Leandro Fernandes Malloy-Diniz (BELO HORIZONTE, MG) Lecio Figueira Pinto (SÃO PAULO, SP) Leila Maria Cardao Chimelli (RIO DE JANEIRO, RJ) Leonardo Ferreira Caixeta (GOIÂNIA, GO) Leticia L. Mansur (SÃO PAULO, SP) Lilian Schafirovits Morillo (SÃO PAULO, SP) Lilian Cristine Hübner (PORTO ALEGRE, RS) Luiz Roberto Ramos (SÃO PAULO, SP) Magali Lourdes Caldana (BAURU, SP) Márcia Lorena Fagundes Chaves (PORTO ALEGRE, RS) Márcia Maria Pires Camargo Novelli (SÃO PAULO, SP) Marcia Radanovic (SÃO PAULO, SP) Marco Antonio Lopes (CATANDUVA, SP) Maria Cecilia de Moura (SÃO PAULO, SP) Maria de Jesus Gonçalves (NATAL, RN) Maria Irma Seixas Duarte (SÃO PAULO, SP) Maria Isis Marinho Meira (SÃO PAULO, SP) Maria Lúcia Gurgel da Costa (RECIFE, PE) Maria Lucia Lebrão (SÃO PAULO, SP) Maria Paula Foss (RIBEIRÃO PRETO, SP) Maria Silvia Carnio (SÃO PAULO, SP) Mariana Kneese Flaks (SÃO PAULO, SP) Marina Martins Pereira Padovani (SÃO PAULO, SP) Marisa Tomoe Hebihara Fukuda (RIBEIRÃO PRETO, SP) Marleide da Mota Gomes (RIO DE JANEIRO, RJ) Melissa Catrini da Silva (SALVADOR, BA) Mirna Lie Hosogi Senaha (SÃO PAULO, SP)	Eliasz Engelhardt (RIO DE JANEIRO, RJ) Elizeu Coutinho de Macedo (SÃO PAULO, SP) Fábio Henrique de Gobbi Porto (SÃO PAULO, SP) Flavia Heloisa Santos (BAURU, SP) Florindo Stella (RIO CLARO, SP) Francelise Pivetta Roque (NOVA FRIBURGO, RJ) Gabriela Coelho Pereira de Luccia (CUIABÁ, MT) Gisela Tinone (SÃO PAULO, SP) Gladys E. Maestre (NEW YORK, USA) Helenice Charchat-Fishman (RIO DE JANEIRO, RJ) Hélio Afonso Ghizoni Teive (CURITIBA, PR) Hélio R. Gomes (SÃO PAULO, SP) Henrique Ballai Ferraz (SÃO PAULO, SP) Isabel Carvalho (PORTO ALEGRE, RS) Ivete Berkenbrock (CURITIBA, PR) Jaci Perissinoto (SÃO PAULO, SP) Jacqueline Abrisqueta Gomes (SÃO PAULO, SP) Jairo Degenszajn (SÃO PAULO, SP) Jed Meltzer (TORONTO, CANADA) Jerson Laks (RIO DE JANEIRO, RJ) Joana Bisol Balardin (PORTO ALEGRE, RS) João Carlos Papaterra Limongi (SÃO PAULO, SP) João Eliezer Ferri-de-Barros (SÃO JOSÉ DOS CAMPOS, SP) José Ibiapina Siqueira Neto (FORTALEZA, CE) Jose Luiz Dias Gherpelli (SÃO PAULO, SP) Mônica Yassuda (SÃO PAULO, SP) Nadia Shigaeff (SÃO PAULO, SP) Natalia Sierra Sanjurjo (BUENOS AIRES, ARGENTINA) Patricia Rzezak (SÃO PAULO, SP) Paula Villela Nunes (SÃO PAULO, SP) Péricles de Andrade Maranhão Filho (RIO DE JANEIRO, RJ) Regina Miksian Magaldi (SÃO PAULO, SP) Renata Areza-Fegyveres (SÃO PAULO, SP) Renata Ávila (SÃO PAULO, SP) Ricardo Allegri (BUENOS AIRES, ARGENTINA) Ricardo Nitrini (SÃO PAULO, SP) Rodrigo Rizek Schultz (SÃO PAULO, SP) Rogerio Gomes Beato (BELO HORIZONTE, MG) Sergio Luís Blay (RIO DE JANEIRO, RJ) Silvia Adriana Prado Bolognani (SÃO PAULO, SP) Simone dos Santos Barreto (NOVA FRIBURGO, RJ) Sonia Maria Dozzi Brucki (SÃO PAULO, SP) Suzana Schonwald Suzana (PORTO ALEGRE, RS) Swathi Kiran (BOSTON, MA) Tânia Maria da Silva Novaretti (MARÍLIA, SP) Maria Teresa Carthery Goulart (SANTO ANDRÉ, SP) Thaís Bento Lima da Silva (SÃO PAULO, SP) Thomas H. Bak (EDINBURGH, UK) Tíbor Rilho Perroco (SÃO PAULO, SP) Uzma Urooj (TORONTO, CANADA) Valeria Santoro Bahia (SÃO PAULO, SP) Vanderci Borges (SÃO PAULO, SP) Vera Lucia Duarte Vieira (SÃO PAULO, SP) Veronique Agnes Guernet Steiner (SÃO PAULO, SP) Vitor Tumas (RIBEIRÃO PRETO, SP) Viviane Amaral Carvalho (BELO HORIZONTE, MG)

